# Supplementary material for: Attitudes of people living with dementia and their carers towards the use of generative artificial intelligence to inform structured medication reviews
Source: Age Ageing. 2026 Nov 2;55(11):afag320. doi: 10.1093/ageing/afag320 (PMC13725756; doi:10.1093/ageing/afag320)
Supplement: Supplementary file 1 [file supplementary_material_afag320.zip › Supplementary_materials_afag320_Appendix 2.docx]

**Attitudes of People Living with Dementia and their Carers towards the use of Generative Artificial Intelligence to inform Structured Medication Reviews**

**The Consolidated Criteria for Reporting Qualitative Research (COREQ): 32-item checklist**

| **No. Item** | **Guide questions/description** | **Notes** |
| --- | --- | --- |
| **Domain 1: Research team and reflexivity** | | |
| **Personal Characteristics** | | |
| 1. Interviewer/facilitator | Which author/s conducted the interview or focus group? | BL conducted the individual interviews and facilitated the focus groups, with JD or JG as co-facilitators for the focus groups. |
| 2. Credentials | What were the researcher's credentials? E.g. PhD, MD | BL, JD, NT, SS, and JG: PhD |
| 3. Occupation | What was their occupation at the time of the study? | JD, NT, SS, and JG are DEM COMM research fellows. BL joined the project as a research assistant. |
| 4. Gender | Was the researcher male or female? | BL, JD, and JG were female. NT and SS were male. All direct contact with participants during data collection was conducted by female researchers. |
| 5. Experience and training | What experience or training did the researcher have? | BL, JD, JG and NT have experience in dementia, qualitative and mixed-methods research; SS has experience in dementia and multimorbidity research. JG additionally has a background in pharmacy. |
| **Relationship with participants** | | |
| 6. Relationship established | Was a relationship established prior to study commencement? | Participants had not met the interviewers prior to the study. Optional pre-interview calls were offered to familiarise participants with the process and discuss accessibility needs or adjustments. |
| 7. Participant knowledge of the interviewer | What did the participants know about the researcher? e.g. personal goals, reasons for doing the research | Participants were informed about the purpose of the research during recruitment and the consent process. The researcher also introduced the study and its purpose at the beginning of each interview or focus group. No participants had met the interviewers prior to the study. |
| 8. Interviewer characteristics | What characteristics were reported about the interviewer/facilitator? e.g. Bias, assumptions, reasons and interests in the research topic | The research team shared an interest in in the use of AI to support structured medication reviews for people living with dementia. The wider project involved the development of an AI-assisted medication review tool. |
| **Domain 2: study design** | | |
| **Theoretical framework** | | |
| 9. Methodological orientation and Theory | What methodological orientation was stated to underpin the study? e.g. grounded theory, discourse analysis, ethnography, phenomenology, content analysis | The study adopted a qualitative design underpinned by an interpretive theoretical position. Data were analysed using inductive reflexive thematic analysis. |
| **Participant selection** | | |
| 10. Sampling | How were participants selected? e.g. purposive, convenience, consecutive, snowball | Participants who responded to the advertisements and met the eligibility criteria were recruited using convenience sampling. |
| 11. Method of approach | How were participants approached? e.g. face-to-face, telephone, mail, email | Carers were recruited through the NIHR People in Research service. PLwD were recruited through advertisements in newsletters of four support services for PLwD. |
| 12. Sample size | How many participants were in the study? | Twenty-six participants took part in the study, comprising 12 PLwD and 14 carers. The 14 carers participated in two focus groups, with seven participants in each group, while the 12 PLwD participated in individual interviews. |
| 13. Non-participation | How many people refused to participate or dropped out? Reasons? | All participants who provided consent completed the interview or focus group, with no withdrawals. |
| **Setting** | | |
| 14. Setting of data collection | Where was the data collected? e.g. home, clinic, workplace | All data collection was conducted remotely via Microsoft Teams or telephone. Face-to-face participation was offered, but all participants elected to participate remotely. |
| 15. Presence of non-participants | Was anyone else present besides the participants and researchers? | No other individuals were present during the interviews or focus groups apart from the participants and researchers. |
| 16. Description of sample | What are the important characteristics of the sample? e.g. demographic data, date | The sample comprised 12 PLwD and 14 carers from different regions of the UK. PLwD were generally older, whereas carers had a relatively younger age profile. The overall sample was predominantly male. PLwD were predominantly White, while carers represented more diverse ethnic backgrounds. The sample also included a range of dementia diagnoses. Further participant characteristics are presented in Table 1. |
| **Data collection** | | |
| 17. Interview guide | Were questions, prompts, guides provided by the authors? Was it pilot tested? | The focus groups and interviews followed the same semi-structured interview guide (Supplementary Appendix 1). The guide was informed by two workshops involving ten healthcare professionals from various backgrounds and PPIE representatives. The interview guide was not formally pilot tested. |
| 18. Repeat interviews | Were repeat interviews carried out? If yes, how many? | No repeat interviews were conducted. |
| 19. Audio/visual recording | Did the research use audio or visual recording to collect the data? | All interviews and focus groups were audio-recorded with permission of participants. |
| 20. Field notes | Were field notes made during and/or after the interview or focus group? | BL kept a post-interview reflexivity diary after each session. Notes were recorded on:   - participant engagement and capacity to consent - self-reflections on interview technique (e.g. how to minimise bias) - reflections of how to refine the interview guide/terminology used for future interviews - initial impressions around possible themes/findings |
| 21. Duration | What was the duration of the interviews or focus group? | The two carer focus groups lasted approximately 1.5 hours each. Individual interviews with PLwD lasted up to 60 minutes. |
| 22. Data saturation | Was data saturation discussed? | Sample size was guided by the concept of information power, with recruitment continuing until further interviews added little new information relevant to the study aims. |
| 23. Transcripts returned | Were transcripts returned to participants for comment and/or correction? | Transcripts were not returned to participants for comment or correction. |
| **Domain 3: analysis and findings** | | |
| Data analysis | | |
| 24. Number of data coders | How many data coders coded the data? | BL, JD and JG independently analysed three transcripts and discussed their initial interpretations. BL coded the remaining transcripts and constructed the initial themes. The three researchers subsequently met to develop and refine the themes. |
| 25. Description of the coding tree | Did authors provide a description of the coding tree? | No coding tree was reported. |
| 26. Derivation of themes | Were themes identified in advance or derived from the data? | Themes were constructed inductively from the data. |
| 27. Software | What software, if applicable, was used to manage the data? | NVivo was used to manage and code the qualitative data. |
| 28. Participant checking | Did participants provide feedback on the findings? | Participants did not provide feedback on the findings. |
| **Reporting** | | |
| 29. Quotations presented | Were participant quotations presented to illustrate the themes / findings? Was each quotation identified? e.g. participant number | Participant quotations are presented throughout the findings to illustrate the analysis. Quotations are identified by participant number and participant group, with P1–P14 representing carers and P15–P26 representing PLwD. |
| 30. Data and findings consistent | Was there consistency between the data presented and the findings? | All themes were developed from the data and supported by illustrative participant quotations. |
| 31. Clarity of major themes | Were major themes clearly presented in the findings? | All themes were developed from the data and supported by illustrative participant quotations. |
| 32. Clarity of minor themes | Is there a description of diverse cases or discussion of minor themes? | Diverse and contrasting perspectives were reported, including differing views on the disclosure of AI use and both positive and cautious perspectives on the use of AI in SMRs. |
